# Elements of Surgical Diagnosis

**Published:** 1886-10

**Authors:** 


ELEMENTS OF SURGICAL DIAGNOSIS. *By* A. PEARCE GOULD, M. S., M. B., *Lond.*, F. R. C. S., *Eng.; Assistant Surgeon to Middlesex Hospital, etc. Large 16mo, pp. 584. Philadelphia:* HENRY C. LEA'S SON & CO. *Chicago:* A. C. MCCLURG & CO.

This effort presents in a concise form the symptoms of many surgical diseases, and groups together those diseases which have certain symptoms in common, pointing out the method of making the differential diagnosis between them. Its chief value lies in its plan of arrangement, making it a practical ready-reference book for refreshing the memory of the young practitioner or advanced medical student. The points made by the author are based upon a sound knowledge of the principles and practice of surgery.

The writer deserves credit for the systematic presentation of the various subjects. F. C. S.

